# Five-hub General Conceptual Framework to improve the vaccination coverage for newly arrived migrants

**DOI:** 10.1093/eurpub/ckac131.032

**Published:** 2022-10-25

**Authors:** ME Tosti, G Marchetti, S Scarso, F D'Angelo, ML Russo, M Marceca, P Karnaki, N Papaevgeniou, S Declich

**Affiliations:** 1 National Centre for Global Health, Italian National Institute of Health, Rome, Italy; 2 Italian Society for Migration Medicine, Rome, Italy; 3 Department of Public Health and Infectious Diseases, Sapienza University of Rome, Rome, Italy; 4 Institute of Preventive Medicine, Environmental and Occupational Health Prolepsis, Athens, Greece

## Abstract

**Background:**

Within the Project “Increased Access for Newly Arrived Migrants-AcToVax4NAM” (Grant n 101018349, 3rd EU Health Programme), a General Conceptual Framework (GCF) was developed for understanding how to improve vaccination coverage for Newly Arrived Migrants (NAM), by characterizing and critically analysing system barriers and possible solutions to increase vaccination

**Methods:**

A logical pathway was hypothesized based on conceptual hubs in the immunization process. The identification of barriers and solutions was carried on by: a) non-systematic revision of scientific and grey literature, institutions and relevant websites, and documents suggested by Consortium Partners; b) qualitative research conducted in each Consortium Country. The GCF was used as a guide for the above mentioned activities and organize results into the GCF itself to enrich it with content

**Results:**

5 conceptual hubs were identified: ENTITLEMENT to vaccination, REACHABILITY of people to be vaccinated, ADHERENCE to vaccination, ACHIEVEMENT of vaccination, EVALUATION of the intervention. All hubs are linked sequentially, starting with Entitlement without which the process cannot take place. Hubs are connected: if vaccination does not take place, it’s important to go back to the previous hubs to understand the barriers. Reachability-Adherence-Achievement are closely related because some approaches are cross-cutting, such as proximity interventions which, in addition to allowing the system to approach NAM, promote adherence and thus possibility of completing the process. Other strategies may be implemented with different purposes: training aims to foster a culturally competent approach to facilitate adherence and avoid vaccination hesitancy, but also to improve competence in the entire process and lead to vaccination completion

**Conclusions:**

The proposed GCF facilitates identification of barriers and possible solutions to the effective achievement of immunization, at all stages of the process

**Key messages:**

• The GCF can be the basis for the creation of country-specific flow-charts through which to test strategies aimed at increasing immunization coverage in NAM.

• The GCF will be useful at EU level, to facilitate both the harmonisation of approaches and interventions and the evaluation of comparable approaches.

